# Effect of Temperature on the Removal of Interferences in the Voltammetric Procedure for the Determination of Cr(VI)

**DOI:** 10.3390/ma17133050

**Published:** 2024-06-21

**Authors:** Malgorzata Grabarczyk, Cecylia Wardak

**Affiliations:** Department of Analytical Chemistry, Institute of Chemical Sciences, Faculty of Chemistry, Maria Curie-Sklodowska University, 20-031 Lublin, Poland; cecylia.wardak@mail.umcs.pl

**Keywords:** surface active substances, chromium determination, adsorptive stripping voltammetry, environmental waters

## Abstract

The aim of this paper was to investigate the effect of temperature on the removal efficiency of surfactant-induced interferences. Surfactants were removed as a result of mixing with XAD-7 resin. The study was carried out using the example of Cr(VI) determination by adsorption stripping voltammetry (AdSV). Measurements were carried out using a solution containing Cr(VI), acetate buffer (pH = 6.2), DTPA, KNO_3_, and different surfactants. Ten mL of the solution was mixed with 0.5 g of XAD-7 resin at different temperatures for 5 min prior to voltammetric measurement. The effect of the mixing temperature of the sample with the resin on the voltammetric Cr(VI) signal in the presence of different surfactants was studied in the range from 20 to 60 °C. The proposed method of removing interference from surfactants by mixing the sample with the XAD-7 resin at 60 °C was used for the determination of trace amounts of Cr(VI) in river water containing non-ionic, anionic, cationic surfactants, and biosurfactants.

## 1. Introduction

Contaminants in environmental water samples can have different chemical characteristics and, in a preliminary classification, they can be roughly divided into organic, inorganic, and metal species pollutants [1,2]. Monitoring each of them is necessary for full control and estimation of the quantity of natural water samples. Unfortunately, the determination of one of them is often disturbed by another. In the case of metal species determination, there is a wide range of methods dedicated to this issue. Commonly used analytical methods for the analysis of trace metals include atomic absorption spectrometry (AAS), inductively coupled plasma optical emission spectroscopy (ICP-OES), and inductively coupled plasma mass spectrometry (ICP-MS). These methods have many advantages but also limitations, and one of them is the high cost of the equipment and, therefore, limited availability [3]. A good alternative to these methods is electrochemical methods, especially voltammetry, which has many advantages, including relatively low cost of the equipment, high sensitivity of the determinations, which makes it possible to achieve very low detection limits, and the possibility of easy modification of the measurement system to meet the requirements of specific determinations [4,5,6]. Unfortunately, voltammetric methods also have limitations, the main one being their sensitivity to surfactants present in the environmental samples. Surfactants often cause a reduction or complete disappearance of signals coming from metal species. This is primarily related to the adsorption of surface-active substances on the surface of the working electrode, thereby reducing or preventing access to the metal species on the electrode surface [7,8,9]. This is a very serious problem, especially since the production and use of surfactants have been growing consistently in recent years, and their concentration in environmental water samples has increased [10,11,12]. Unfortunately, there are only a few procedures described in the literature dedicated to eliminating interference from surface-active substances in voltammetric determinations. In the vast majority of them, preliminary mineralization of the samples by, e.g., UV irradiation or microwave heating before determination was recommended [13,14,15,16]. As is known, the sample mineralization process is time-consuming and requires the use of additional equipment, which may cause changes in the speciation of the analyzed metal ions in the sample, leading to falsified results. Less invasive methods designed to eliminate interference in voltammetric measurements related to the presence of surfactants in the sample include pre-mixing the sample with colloidal silica or, above all, Amberlite XAD-7 resin. Particularly good results are achieved when the sample is mixed with XAD-7 resin; during mixing, surfactants are adsorbed on the resin, while the metal ions being determined remain in solution. Amberlite XAD-7 resin is a polymeric sorbent derived from acrylic esters, which can sorb hydrophobic compounds from water as well as hydrophilic compounds from organic solvents and is used to purify water from organic pollutants (dyes, pesticides, and detergents) [17]. Therefore, It is not surprising that this method of reducing interference related to the presence of surfactants has been used in the voltammetric analysis of many elements [18,19,20,21,22]. The main difficulty is to select appropriate conditions, mainly the composition and pH of the basic electrolyte, to ensure the highest possible efficiency in the adsorption of surface-active substances on the resin and at the same time prevent the adsorption of metal ions on the resin in such conditions. Although the resin mixing procedure ensures a reduction in interference from surface-active substances, there is still a need to further improve the effectiveness of removing these interferences. In the procedures described in the literature using the adsorption properties of the XAD-7 resin to eliminate interference, the initial mixing of the sample with the resin was carried out at room temperature. The aim of our research was to investigate whether and how changing the temperature in the range of 20 to 60 °C in this process can increase the efficiency of removing interferences related to the presence of surface-active substances. These studies were carried out on the determination of Cr(VI) by adsorption stripping voltammetry (AdSV), for which preliminary mixing of the sample with Amberlite XAD-7 resin was used to obtain satisfactory results [23,24]. The choice of Cr(VI) for research is also supported by the fact that chromium speciation analysis is one of the most important and most frequently performed speciation analyses. This is mainly related to the drastically different toxicities of the two most popular forms of this element in the environment. Cr(III) is considered to be an essential nutrient, unlike Cr(VI) which is carcinogenic and toxic [25,26,27]. Despite widespread awareness of the toxicity of Cr(VI), chromium is widely used and the demand for its compounds is constantly growing. Chromium and its compounds are widely used in the industry; they are used, among other things, in the production of stainless steel, in galvanization processes, for the production of glass and cement, in the cosmetics industry, in dietetics, and as dyes. For this reason, there is a need for highly selective procedures that enable the determination of Cr(VI) in environmental samples, such as surface waters containing high concentrations of surfactants. In voltammetry, the selection of the electrode on which the accumulation of the substance to be determined occurs is crucial. The first voltammetric procedure was carried out using a mercury hanging drop electrode (HMDE). As awareness of mercury’s toxicity increased, the search for other electrode materials began. Currently, many new materials and design solutions have been described in the literature, but they have not successfully replaced HMDE [28,29,30] in all cases. Such an example is the determination of Cr(VI). There are reports in the literature on the use of a bismuth electrode for the determination of Cr(VI) by adsorption stripping voltammetry, but the best results are still obtained using mercury electrodes [31,32,33]. Therefore, the procedure proposed in this work uses a refreshable mercury film silver-based electrode. This electrode provides very good results comparable to the mercury electrode, but its design and the small amount of mercury placed in the seal guarantee safety during its use [34,35].

## 2. Materials and Experimental Work

### 2.1. Reagents

Each of the chemicals employed was of analytical reagent grade. For all purposes, distilled water obtained from a Milli-Q water purification system (Millipore, Livingston, UK) was used. Stock standard solutions of Cr(VI) as K_2_CrO_4_ and Cr(III) as CrCl_3_ with concentrations of 1 g L^−1^. CH_3_COOH, KNO_3_, and Suprapure NaOH were obtained from Merck (Darmstadt, Germany). Acetate buffer contained 0.5 mol L^−1^ of acetic acid and was adjusted to pH = 6.2 with sodium hydroxide. KNO_3_ was additionally purified by recrystallization. Diethylenetriaminepentaacetic acid (DTPA) was obtained from Sigma (St. Louis, MO, USA). DTPA solution contained 0.2 mol L^−1^ of diethylenetriaminepentaacetic acid dissolved at pH > 7 in the presence of sodium hydroxide and was adjusted to pH = 6.2 with acetic acid. Triton X-100, sodium dodecyl sulfate (SDS), cetyltrimethylammonium bromide (CTAB), and rhamnolipids were acquired from Fluka (Buchs, Switzerland). Amberlite XAD-7, acquired from Sigma, was washed four times using distilled water and dried at a temperature of 50 °C.

### 2.2. Equipment

All voltammetric experiments were performed using a µAutolab (Eco Chemie, Utrecht, The Netherlands) connected to a personal computer operated by GPES 4.9 software. A 10 mL thermostated voltammetric quartz cell was used. The electrode stand consisted of a refreshable mercury film silver-based electrode (Hg(Ag)FE) as the working electrode, an Ag/AgCl electrode filled with saturated NaCl as the reference electrode, and a platinum counter electrode. The construction and procedure of the surface refreshing of Hg(Ag)FE are precisely described in detail [34,35]. The Hg film area was 7 mm^2^. The analyzed solutions were thermostated using a TIP 200 laboratory thermostat (WSL, Poznan, Poland), with a temperature stability of ± 0.1 °C. The pH values of the solutions were measured using a CP-401 pH meter (Elmetron, Zabrze, Poland).

### 2.3. Measumerent Procedure

For laboratory tests, a synthetic sample containing a fixed concentration of Cr(VI) and various concentrations of surfactants, 0.04 mol L^−1^ acetate buffer (pH = 6.2), 0.01 mol L^−1^ DTPA, 0.5 mol L^−1^ KNO_3_, and 0.5 g of XAD-7 were prepared. Every sample was placed into the thermostated voltammetric cell; before voltammetric measurement, it was deaerated by passing nitrogen gas through it for 5 min via stirring. During 5 min of stirring, the deoxygenation of the solution and removal of organic matter via adsorption onto the XAD-7 resin occurred simultaneously. During this time, the sample was thermostated at the desired temperature in the range of 20 to 60 °C in 5 °C increments.

### 2.4. Course of Voltammetric Measurement

To start the voltammetric measurement, the silver wire of the Hg(Ag)FE electrode was pushed out of the electrode body containing mercury into the solution being analyzed. The previously existing wire was covered with a new mercury film. Voltammetric measurements were taken in two steps. In the first step, a potential of −1.0 V was applied to Hg(Ag)FE for 30 s, which took place while mixing the solution. During this time, Cr(VI) was reduced to Cr(III) and, after forming a complex with DTPA as a Cr(III)-H_2_DTPA electrochemical active complex, it was adsorbed onto the electrode surface. Then, stirring was stopped and the solution was left to settle for 5 s. In the second step, voltammetric stripping was performed in different pulse modes in the potential range from −1.0 to −1.4 V at a scan rate of 20 mV s^−1^ and an amplitude of −50 mV. The voltammogram showed a peak associated with the reduction of Cr(III) to Cr(II), enhanced by the catalytic effect resulting from cyclic oxidation and subsequent reduction due to the presence of KNO_3_. The intensity of the peak current was directly proportional to the concentration of Cr(VI) in the solution and was used for quantitative analysis. Under such conditions, the calibration graph for Cr(VI) for an accumulation time of 30 s was linear, ranging from 1 × 10^−9^ to 1 × 10^−7^ mol L^−1^, and obeyed the equation y = 101.2x + 17.5, where y and x were the peak current (nanoampere) and Cr (VI) concentration (nanomol per liter), respectively. The linear correlation coefficient was r = 0.998. The detection limit was equal to 2.4 × 10^−10^ mol L^−1^.

## 3. Results and Discussion

### 3.1. Speciation and the Influence of Temperature on Cr(VI) Voltammetric Signal

Chromium analysis is one of the most important analyses in speciation analysis. In the case of Cr(VI) determination, a very important issue is the possibility of determining the presence of the highest possible excess of Cr(III) ions. There are many ways of masking Cr(III), but the most popular is to exploit the difference in the electrochemical activity of chrome complexes with DTPA. For the determination of Cr(VI) in the presence of Cr(III), Boussemart et al. [36] took advantage of the fact that the analytical signal corresponding to Cr(III) present in the solution decreased with time after the addition of DTPA. This is due to the fact that the initially formed electrochemically active Cr(III)-DTPA complex transitions to an inactive one over time. In the case of Cr(VI) present in the solution, the complex with DTPA is only formed during the accumulation step, so the timing of DTPA addition does not affect the analytical signal corresponding to Cr(VI). As demonstrated in the work [37], the rise in temperature accelerates the transition of Cr(III)-DTPA complexes to an electrochemically inactive form and, at 40 °C, a 5 min time allows the determination of Cr(VI) in the presence of up to 100-fold excess Cr(III) without interference.

It was investigated whether increasing the temperature of the solution from which the voltammetric measurement was carried out affected the magnitude of the Cr(VI) signal. For this purpose, a solution was prepared containing 5 × 10^−8^ mol L^−1^ Cr(VI), 0.04 mol L^−1^ acetate buffer (pH = 6.2), 0.01 mol L^−1^ DTPA, and 0.5 mol L^−1^ KNO_3_, and the measurements were carried out as described in Section 2.4 (potential and accumulation time −1.4 V 30 s; signal recording ranged from −1.0 V to −1.4 V) at different temperatures ranging from 20 °C to 60 °C in 5 degree steps. It turned out that throughout the entire temperature range tested, the chromium signal remained at the same level with a relative standard deviation of 3.1%.

### 3.2. Surfactants

Surfactants are a group of chemical compounds whose molecules are made up of two elements with opposite affinities for water: a hydrophobic element (with a low affinity for water) and a hydrophilic element (with a high affinity for water). Surfactants are mainly used in the form of aqueous solutions and are therefore classified according to the type of hydrophilic group. A distinction is made between anionically active, cationically active, amphoteric, and non-ionic compounds. We can also distinguish between biosurfactants, which, unlike synthetic surfactants, are obtained through microbial biosynthesis processes, mainly using bacteria and yeast. Biosurfactants are characterized by greater stability at extreme temperatures, high pH, different salinity, low toxicity, and biodegradability, which gives them an advantage over their chemical counterparts.

Due to their properties, surfactants are used as wetting agents and emulsifiers; as the primary active ingredient in washing, detergent, and cleaning agents; and as auxiliary agents, e.g., in the textile, leather, and plastics industries. Cationic active compounds and certain amphoteric compounds show strong bactericidal properties and are therefore used, among other things, for disinfection. Due to this widespread use, they enter the environment in large quantities. When analyzing aqueous environmental samples, it is essential to take into account the presence of surfactants in them. For the research carried out in this thesis, one representative of each of the surfactants discussed earlier, namely Triton X-100, SDS, CTAB, and rhamnolipids, was selected. Information on these surfactants is provided in Table 1.

### 3.3. Effect of Temperature on Removal of Interference from Surfactants

As demonstrated in earlier work, a very good way to reduce the interferences occurring in the voltammetric determination of Cr(VI) determination associated with the presence of surfactants is to mix the sample with the XAD-7 resin [23,24]. In this procedure, measurements were carried out at 40 °C in order to increase selectivity in the speciation analysis of chromium, as mentioned in Section 3.1. However, it was not investigated whether increasing the temperature could affect the efficiency of the surfactant removal by the resin. Therefore, the primary objective of our study was to investigate in detail the effect of temperature on the Cr(VI) signal in the presence of different concentrations of different surfactants by mixing with the XAD-7 resin. For this study, the following surfactants were used: TritonX-100, SDS, CTAB, and rhamnolipid, representing non-ionic surfactants, anionic, cationic, and biosurfactants, respectively.

#### 3.3.1. Triton X-100 Nonionic Surfactant

Triton X-100 is a non-ionic surfactant (uncharged surfactant), which is a liquid that is well-soluble in water at room temperature. Chemically, it is a mixture of octylphenol ethoxylates commonly available in the market. Triton X-100 has been recognized as a standard among similar products, widely used in many commercial and industrial products. It is used as a substrate in cleaning agents and in the production of textiles and fibers, and it is also commonly used as an emulsifier in artists’ colors. The areas of application include the purification and extraction of proteins, which are also components of biological buffers. Such widespread use is due to the fact that Triton X-100 has excellent detergent properties, excellent wetting ability, and excellent grease and oil removal from hard surfaces. Due to such widespread use, Triton X-100 is released into the environment in relatively large quantities. The biodegradation of Triton X-100 is problematic, and the degradation product is considered ecotoxic because it has hormone-like properties and thus poses a potential danger to flora and fauna. Taking this into account, Triton X-100 was chosen to study the effect of temperature on the removal of interferences associated with it by mixing with the XAD-7 resin.

**Figure 1:**
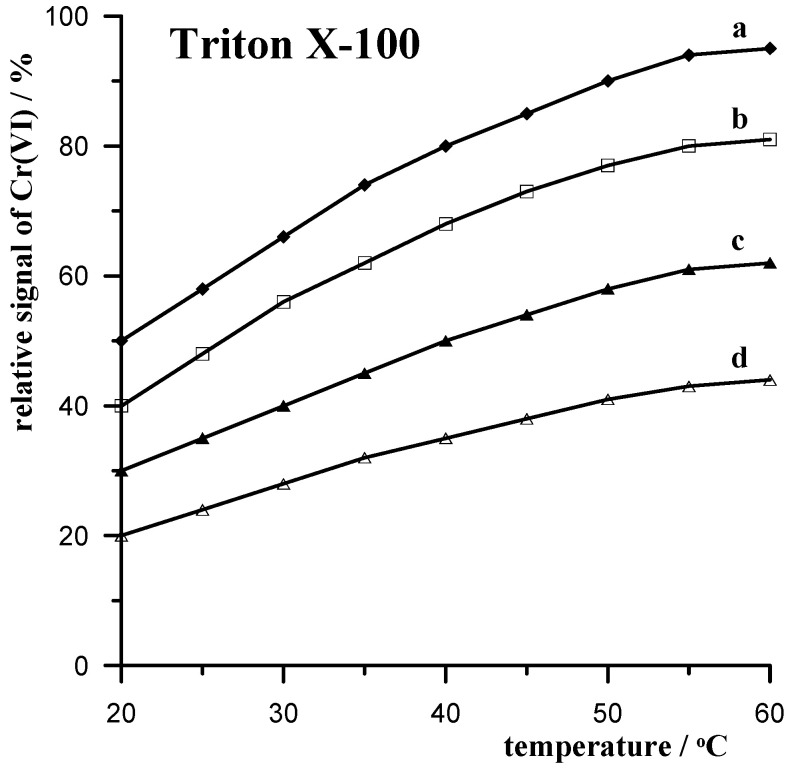
Temperature dependence of the relative 5 × 10^−8^ mol L^−1^ Cr(VI) signal when mixing a 10 mL sample with 0.5 g of XAD-7 resin in the presence of the following concentrations of Triton X-100: 10 mg L^−1^ (a); 15 mg L^−1^ (b); 20 mg L^−1^ (c); 25 mg L^−1^ (d).

The effect of Triton X-100 on the analytical signal of chromium in different temperatures was examined for solutions containing 0.04 mol L^−1^ acetate buffer (pH = 6.2), 0.01 mol L^−1^ DTPA, 0.5 mol L^−1^ KNO_3_, and 0.5 g of XAD-7. Experiments were carried out using four different concentrations of Triton X-100 (10, 15, 20, and 25 mg L^−1^) at temperatures varying from 20 to 60 °C at a frequency of 5 °C. The obtained results are presented in Figure 1, which shows the dependence of the relative chromium signal as a function of the temperature at which the sample was mixed with the resin. The relative signal was determined in relation to the chromium signal recorded in the absence of Triton X-100, whose value was taken as 100%. As can be seen, the chromium signal increases with increasing temperature ranging from 20 to 60 °C, at which point the sample is mixed with the resin and approaches the value obtained in the absence of Triton X-100. However, at temperatures above 50 °C, this increase is already negligible.

#### 3.3.2. CTAB Cationic Surfactant

Cetyltrimethylammonium bromide (CTAB) is a quaternary ammonium surfactant with the structural formula [(C_16_H_33_)N(CH_3_)_3_]Br. It is an effective antiseptic against bacteria and fungi and is therefore used in numerous antibacterial and antiseptic products. CTAB is also used in cosmetics, mainly hair conditioners, soaps, and shampoos, as well as in household products such as fabric softeners. It plays an important role as one of the main ingredients in some DNA extraction buffers. Due to its positive charge, CTAB, like other cationic surfactants, strongly adsorbs onto negatively charged soil and sediment surfaces. Due to these properties and their widespread use, CTAB is expected to be present in many elements of the environment. Therefore, it was CTAB that was chosen to represent positively charged surfactants in our study.

**Figure 2:**
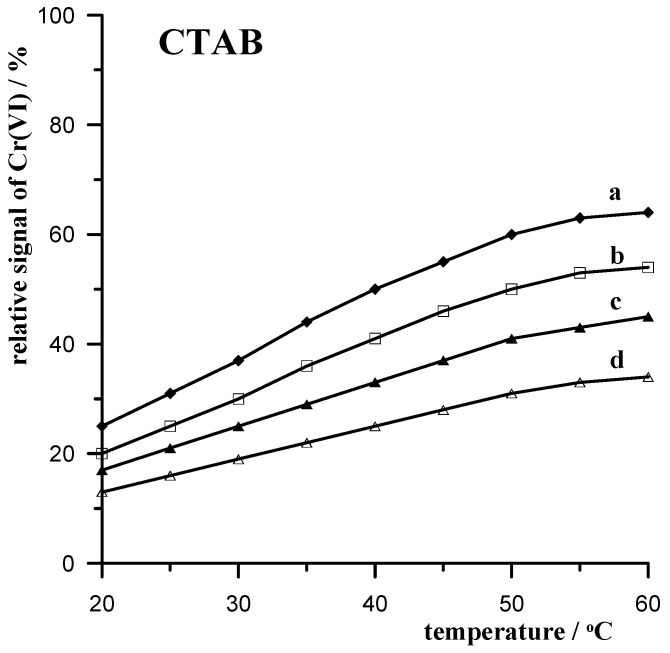
Temperature dependence of the relative 5 × 10^−8^ mol L^−1^ Cr(VI) signal when mixing a 10 mL sample with 0.5 g of XAD-7 resin in the presence of the following concentrations of CTAB: 10 mg L^−1^ (a); 15 mg L^−1^ (b); 20 mg L^−1^ (c); 25 mg L^−1^ (d).

The effect of CTAB on the chromium signal was studied in a manner similar to Triton X-100, depending on the applied mixing temperature of the solution with the XAD-7 resin in order to remove this interference from the solution as much as possible. The results obtained are presented in Figure 2. As can be seen, similarly to Triton X-100, an increase in the mixing temperature with the resin clearly increases the removal efficiency of the CTAB interferent, which is reflected in the increased chromium signal. As with Triton X-100, an initial increase in temperature above 20 °C clearly increases the CTAB removal efficiency of the resin; however, as the temperature approaches 60 °C, this enhancement decreases.

#### 3.3.3. SDS Anionic Surfactant

**Figure 3:**
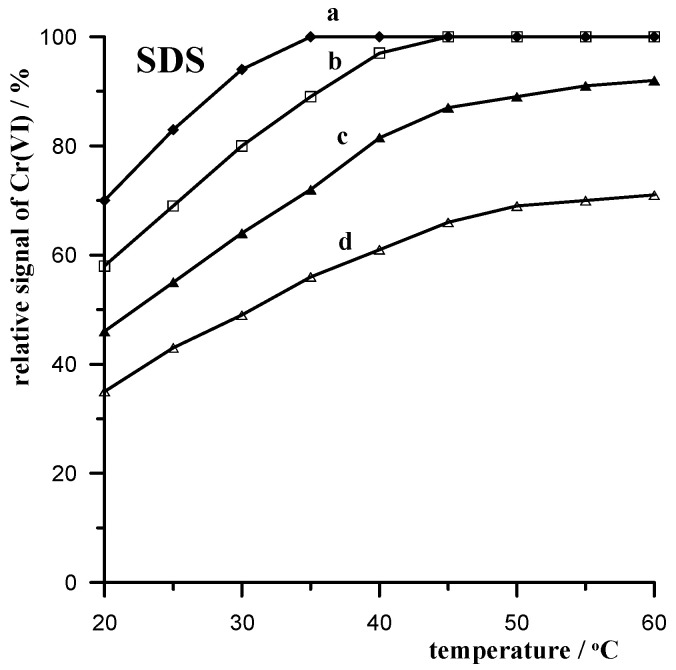
Temperature dependence of the relative 5 × 10^−8^ mol L^−1^ Cr(VI) signal when mixing a 10 mL sample with 0.5 g of XAD-7 resin in the presence of the following concentrations of SDS: 10 mg L^−1^ (a); 15 mg L^−1^ (b); 20 mg L^−1^ (c); 25 mg L^−1^ (d).

Anionic surfactants contain anionic functional groups at their heads, such as sulfates, sulfonates, phosphates, and carboxylates. One of the more common commercially available options is sodium dodecyl sulfate (SDS), with the chemical formula NaC_12_H_25_SO_4_. SDS, like other anionic surfactants, is widely used in industrial as well as household cleaning and pesticide formulations. Anionic surfactants are used as emulsifiers, solubilizers, and wetting agents in the cosmetic industry. They are also used in the textile industry as well as for wastewater treatment. Like other surfactants, SDS, which, according to the literature, is the most widely used surfactant in the world, also ends up in large quantities in the environment. Since the advent of COVID-19, more SDS has been released into the natural aquatic environment. Therefore, it was chosen for our study to investigate the influence of SDS as an interferent in the voltammetric determinations of environmental waters using the Cr(VI) determination as an example. The effect of SDS on the Cr(VI) signal using mixing of the sample with XAD-7 at different temperatures is shown in Figure 3. As can be seen by comparing the effect on the Cr(VI) signal of Triton X-100 and CTAB, it is clear that SDS has a lesser interfering effect. This can be further reduced by increasing the mixing temperature of the resin from 20 to 40 °C. A further increase in temperature to 60 °C reduces the interfering effect of SDS, but to a decidedly lesser extent.

#### 3.3.4. Rhamnolipid Biosurfactant

**Figure 4:**
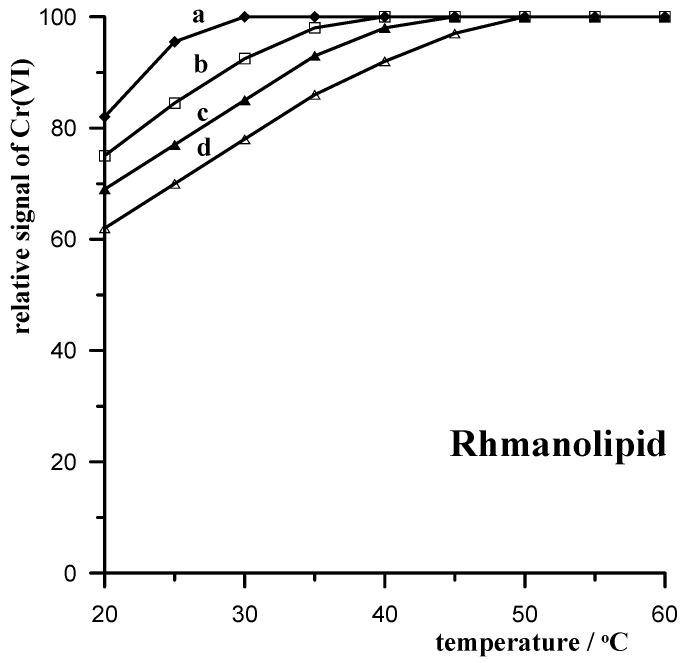
Temperature dependence of the relative Cr(VI) signal when mixing a 10 mL sample with 0.5 g of XAD-7 resin in the presence of the following concentrations of rhamnolipids: 10 mg L^−1^ (a); 15 mg L^−1^ (b); 20 mg L^−1^ (c); 25 mg L^−1^ (d).

As one of the better-known bioproducts, biosurfactants have many applications in the environmental, food, agricultural, petroleum, paper, cosmetic, and pharmaceutical industries. These natural compounds, which are produced by microbial cells, have several advantages over their synthetic counterparts, including high biodegradability, low toxicity, better environmental compatibility, acceptable surface activity at extreme temperatures, pH, and salinity, and the ability to be synthesized from renewable raw materials. The main reason for the current global interest in the production of rhamnolipids is their wide range of advantages, as well as their potential applications in various industries along with their ‘environmentally friendly’ characteristics. Rhamnolipids with a glycolipid-type structure, produced mainly by Pseudomonas aeruginosa, are currently the most intensively studied biosurfactants. Therefore, our research also examined their influence on the voltammetric determination of Cr(VI) in detail. As for the above surfactants, the effect of rhamnolipid on the Cr(VI) signal was examined for samples mixed with the XAD-7 resin at various temperatures ranging from 20 to 60 °C. The obtained results are presented in Figure 4.

It is clear that rhamnolipid has the least interfering effect on the voltammetric determination of Cr(VI), which can be further reduced by mixing with the XAD-7 resin. Also in this case, an increase in the mixing temperature with the resin increases the effectiveness of eliminating interference and the possibility of obtaining an undisturbed chromium signal.

### 3.4. Analysis of Aqueous Environmental Samples for the Presence of Surfactants

Water collected from the Bystrzyca River was used to test the applicability of the developed procedure for Cr(VI) determination in the presence of high concentrations of surfactants. Water samples were enriched with a known concentration of Cr(VI), and their recovery was tested by mixing with the XAD-7 resin at a temperature of 60 °C. The obtained results presented in Table 1 fully confirm that the proposed procedure allows the determination of trace concentrations of Cr(VI) in water rich in surfactants with an RSD not exceeding 5%.

## 4. Conclusions

In light of the increased awareness concerning the trace concentrations of toxic metal ions in environmental matrices, there is a need for procedures that enable their determination in such samples. In the analysis of environmental samples, a very important aspect is the consideration of their matrix, which can have a decisive influence on the results of the determination in the voltammetric method. In the case of the determination of metal ions, the matrix that may interfere with or even prevent the correct voltammetric measurement is primarily surfactants. With their increasing use in everyday life and many industries, these compounds enter the environment in large quantities. As demonstrated in this work, using the example of the determination of toxic Cr(VI) ions, these compounds cause a reduction or complete loss of the analytical chromium signal, and a very good way to avoid these interferences is to pre-mix the sample with the XAD-7 resin. It has also been shown that it is possible to increase the efficiency of the removal of these interferences by increasing the mixing temperature of the sample with the resin from 20 to 60 °C. Surfactants with different charges, such as Triton X-100, SDS, CTAB, and rhamnolipids, were experimentally tested, and in each case, it was found that increasing the mixing temperature of the sample with the resin increased the chromium signal, and this signal increasingly approached the signal value obtained in the absence of interferents.

## Figures and Tables

**Table 1:** Determination of Cr(VI) using the AdSV method in Bystrzyca water samples enriched with 1 × 10^−8^ mol L^−1^ Cr(VI) and different concentrations of selected surfactants. Measurements were carried out by mixing the sample with 0.5 g of XAD-7 resin for 5 min at 60 °C.

Sample	Surfactant Added		Recovery of Cr(VI) (%)	RSD (*n* = 5) (%)
	Type	Concentration (mg L^−1^)		
Bystrzyca river water	Triton X-100	2.5	95.3	3.9
		5	97.1	4.6
	CTAB	1	96.7	4.8
		2.5	94.7	4.2
	SDS	10	98.8	3.7
		15	101.4	3.8
	Rhamnolipid	20	100.7	4.3
		25	98.0	3.3

## Data Availability

The original contributions presented in the study are included in the article, further inquiries can be directed to the corresponding author.
